# Catch and arrest: exploiting the retromer by a Chlamydial effector

**DOI:** 10.1038/sigtrans.2017.39

**Published:** 2017-06-30

**Authors:** Zhao-Qing Luo

**Affiliations:** 1 Purdue Institute for Inflammation, Immunology and Infectious Disease and Department of Biological Sciences, Purdue University, West Lafayette, IN, USA

Three recent papers, including one by Sun *et al.*^1^ in this issue of *Signal Transduction and Targeted Therapy*, analyze the structural basis of the inhibition of retromer function by the Chlamydial effector protein IncE. These studies reveal that IncE recognizes a highly conserved region on SNX5, a component of the retromer, leading to the inhibition of retromer activity and the promotion of bacterial virulence.

The obligate bacterial pathogen *Chlamydia trachomatis* is a major cause of sexually transmitted diseases; it proliferates in host cells by establishing a compartment called inclusion.^2^ The biogenesis of the inclusion requires a large cohort of effector proteins that are translocated into the host cell, presumably by a type III secretion system (T3SS).^2,3^ These effectors modulate various host cell processes by interacting with host proteins, particularly those involved in vesicle trafficking.^2^

More than 50 T3SS effectors have been identified for *C. trachomatis*, most of which share little homology with proteins of known activity, nor do they harbor predictable structural motifs suggestive of potential function.^4^ The lack of activity associated with bacterial effectors predicable by currently available bioinformatics tools is arguably the biggest challenge in functional dissection of these proteins.^2,3^ By affinity purification of effectors transiently expressed in host cells, the potential host targets for more than 30 *Chlamydial* effectors had been identified.^5^ Among these, IncE was found to interact with sorting nexins (SNXs) 5/6, components of the retromer,^5^ which is a complex formed by five different proteins (heteropentamer) that participates in a wide range of cellular processes by mediating retrograde transport of transmembrane cargo from endosomes to destinations such as the *trans*-Golgi compartment.^6^

Deletion analysis revealed that IncE binds the Phox domain of sorting nexin 5 (SNX5-PX), a region involved in binding the signaling lipid phosphatidylinositol 3-phosphate.^5^ The three structural studies showed that this effector specifically recognizes a highly conserved hydrophobic groove on the PX domain of SNX5.^1,7,8^ Strikingly, each of these studies identified a 26-residue region (IncE_107–132_) localized in the carboxyl end of IncE as the site that directly engages SNX5 (Figure 1), highlighting the importance of this cytosolically exposed domain in the recruitment of the host protein. The high-resolution structures of the IncE_107–132_–SNX5_22–170_ complex allowed the identification of several residues on IncE that are directly involved in the interactions via hydrogen bonds, hydrophobic interactions and salt bridges.^1,7,8^ As predicted by the structures, mutations in these residues significantly reduced the binding affinity between IncE and SNX5.^1,7,8^

Interestingly, the site recognized by IncE appears to be the site used by SNX5 to recognize the cation-independent mannose-6-phosphate receptor (CI-MPR), one of the established cargo molecules for the retromer.^1,7,8^ Overexpression of IncE but not its mutants with lower affinity for SNX5 interfered with the transport of CI-MPR to the *trans*-Golgi compartment in mammalian cells. Consistently, IncE mutants with lower affinity for SNX5 can no longer be recruited to the inclusion during infection.^7,8^ As the abundance of effectors translocated into the host cell is often extremely low, these observations suggest that IncE binds SNX5 with an affinity higher than CI-MPR. Thus, the binding of IncE may prevent the recruitment of the CI-MPR cargo molecule by the retromer. Indeed, in cells infected by *C. trachomatis*, the interactions between SNX5 and CI-MPR were disrupted.^7^ Alternatively, IncE binding may disrupt the assembly of the retromer, thus subverting its ability to recruit cargo molecules. Given the high affinity between the 28-residue IncE peptide and SNX5, it is tempting to predict that delivery of this peptide into host cells may interfere with the virulence of *C. trachomatis.*

Interference with the retromer facilitates intracellular replication of *C. trachomatis*,^5^ suggesting that this host machinery functions to restrict the pathogen. Although these structural studies have laid a solid foundation for further analysis of the role of IncE and other effectors in Chlamydial virulence, some important questions remain. For example, is the binding *per se* sufficient for the function of IncE? As a protein of 132 residues predicted to harbor two transmembrane domains,^5^ the likelihood of IncE to confer a biochemical activity may be low but further study to determine the structure of full-length IncE may reveal additional motifs important for its activity. Alternatively, it is possible that the retromer is biochemically attacked by other effectors after being recruited into close proximity of the inclusion by IncE (Figure 1). Similarly, how does the binding of IncE counteract the restriction of intracellular growth of *C. trachomatis* by retromer? The current model suggests that IncE interferes with the interactions between retromer and the endosomes, thus probably blocking the maturation of the inclusion into lysosome.^5^ Given the potential role of the retromer in the regulation of autophagy,^9^ it will be interesting to determine whether IncE affects the maturation of the autophagosome, a process known to restrict intracellular bacterial pathogens.^10^

Structural analyses have contributed significantly in the study of bacterial virulence factors. For example, the AMPylator function of the Legionella effector SidM (also known as DrrA) was revealed by structural analysis,^11^ so was the unconventional E3 ubiquitin ligase activity conferred by a Cys–His–Asp catalytic triad in SidC.^12^ Importantly, SidM has high affinity for its cellular target Rab1,^13^ validating the notion that binding *per se* rarely is the complete picture for the subversion of host functions by virulence factors. As bacterial pathogens often exploit host processes by effectors that execute the reactions with higher efficiencies, functional dissection of these virulence factors will continue to generate new insights into both bacterial pathogenesis and host cell biology. For Chlamydial research, functional elucidation of the scores of its T3SS effectors will produce not only exciting discoveries, but also leads for the development of novel therapeutics against the diseases caused by the bacterium. Efforts from structural biologists will be indispensible in achieving the goal.

## Figures and Tables

**Figure 1:**
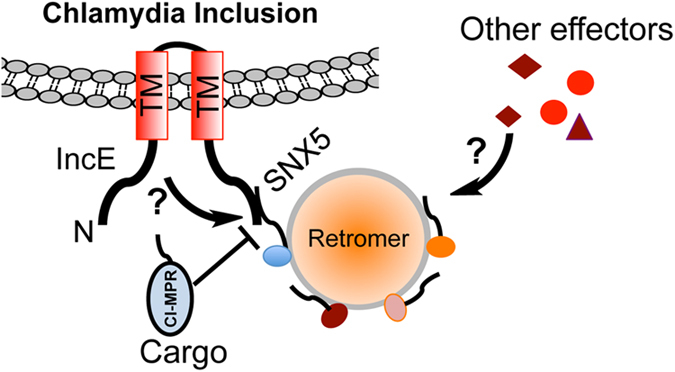
A model for the inhibition of retromer by Chlamydial effector(s). The effector IncE is inserted into the membranes of the inclusion via its two transmembrane motifs in a way that both its amino and carboxyl termini are exposed to the cytoplasm of the host cell. IncE recruits the retromer to the proximity of the inclusion by binding SNX5 with high affinity. The binding to SNX5 by IncE may exclude its association with cargo molecules such as CI-MPR. Binding by IncE may also prevent the retromer from complete assembly or allow further modulation of its activity by additional IncE activity or by other Chlamydial T3SS effectors (arrows with a question mark).
